# Southern African HIV Clinicians Society guidelines for solid organ transplantation in human immunodeficiency virus: An evidence-based framework for human immunodeficiency virus-positive donors and recipients

**DOI:** 10.4102/sajhivmed.v21i1.1133

**Published:** 2020-10-15

**Authors:** Jeremy S. Nel, Francesca Conradie, Jean Botha, Harriet Etheredge, June Fabian, Leon Levin, Ahmad H. Mazanderani, Michelle Moorhouse, Elmi Muller, Caroline Tiemessen, David Thomson, Julia Turner

**Affiliations:** 1 Helen Joseph Hospital, Department of Medicine, University of the Witwatersrand, Johannesburg, South Africa; 2 Clinical HIV Research Unit, Wits Health Consortium, Johannesburg, South Africa; 3 Department of Medicine, University of the Witwatersrand, Johannesburg, South Africa; 4 Helen Joseph Hospital, Johannesburg, South Africa; 5 Wits Donald Gordon Medical Centre, Johannesburg, South Africa; 6 Faculty of Health Sciences, University of Witwatersrand, Johannesburg, South Africa; 7 Right to Care, NGO, Johannesburg, South Africa; 8 Centre for HIV and STIs, National Institute for Communicable Diseases, National Health Laboratory Service, Johannesburg, South Africa; 9 Wits RHI, University of the Witwatersrand, Johannesburg, South Africa; 10 Department of Medicine, University of Cape Town, Cape Town, South Africa; 11 Division of General Surgery, Groote Schuur Hospital, Cape Town, South Africa; 12 DST/NRF Chair of HIV Vaccine Translational Research, Pretoria, South Africa; 13 Right to Care NGO, Centurion, South Africa

## Introduction

These guidelines, intended for transplantation healthcare practitioners in Southern Africa, seek to sketch an evidence-based framework for human immunodeficiency virus (HIV)-positive donors and recipients regarding solid organ transplantation. The guidelines include considerations for the transplantation of organs from HIV-positive donors to HIV-negative recipients. Donor and recipient eligibility, HIV transmission risks and ethical considerations are discussed.

South Africa has over 7 million people living with HIV, the largest such population in the world.^1^ Approximately 20% of people aged 15–64 years are living with HIV in South Africa.^2^ Compared with HIV-negative controls, people living with HIV are at increased risk of end-stage organ disease. Human immunodeficiency virus-positive patients demonstrate a faster decline in renal function than HIV-negative patients, and an approximately threefold increased risk of end-stage renal disease.^3,4,5^ Furthermore, people living with HIV have a higher risk of acute and chronic liver failure, accelerated progression of hepatitis B and C co-infection to cirrhosis, and an increased risk of hepatocellular carcinoma compared with HIV-negative controls.^6,7,8^ The increased risk posed by HIV is multifactorial, with direct HIV toxicities, opportunistic infections, chronic systemic inflammation, immune dysfunction, antiretroviral therapy (ART) side-effects and genetic factors all potentially playing synergistic, contributory roles.^9,10^

In many cases of end-stage renal or liver disease, organ transplantation may offer definitive cure of the underlying condition, with a resultant reduction in mortality. However, there is a critical shortage of available organs. Although thousands of South Africans are waitlisted for transplantation, only approximately 500 solid organ and corneal transplantations are performed each year.^11^ The number of transplantations performed in South Africa has declined over the past decade.^11,12^ A prolonged waiting period prior to organ transplantation adds substantial financial, morbidity and mortality costs to the patients concerned and to the healthcare sector as a whole.

One reason for the shortage of available organs is that potential donors may be excluded if they are HIV-positive, particularly for living donors. Historically, there has been considerable concern about the transmission of HIV and other opportunistic infections from the donor to the recipient, as well as concerns about graft viability. The high prevalence of HIV within the South African population, particularly in persons aged < 65 years, means that a large pool of potential donors may be unlocked if it is possible to utilise organs from this group safely for transplantation.

On the recipient side, it has only recently become understood that HIV-positive patients can make suitable recipients of solid organ transplants because of groundbreaking work both locally and internationally. These guidelines seek to provide best practice recommendations for considering HIV-positive individuals as both potential organ donors and organ recipients and are intended for use by healthcare practitioners in the transplantation field in Southern Africa in both the state and private sectors. Considerations for HIV-negative recipients of organs from HIV-positive donors are also discussed.

## Guideline development process

An expert panel was constituted, consisting of HIV experts from the Southern African HIV Clinicians Society, representatives from the South African Transplant Society, the National Institute for Communicable Diseases, transplant surgeons from the University of Cape Town and Wits Donald Gordon Medical Centre, a medical ethics specialist and a transplant infectious diseases specialist. Both adult and paediatric domains were represented. The scope and outline of the guidelines were discussed at a meeting in November 2018. A PubMed literature search was conducted on all publications relating to the keywords ‘HIV’, ‘transplantation’ and ‘transplant’ up to January 2020. Owing to a paucity of published data, all types of articles were reviewed, including case series and case reports. Draft guidelines were compiled and circulated for comment and amendments to the entire committee prior to publication, and decisions were made by consensus. The guidelines will be reviewed as needed in the light of new evidence.

## Evidence to date

### Human immunodeficiency virus-positive recipients of solid organ transplants

Patients with HIV have received organ transplants since the 1980s, although, owing to inconsistent testing at the time, many of these patients were only diagnosed with HIV months to years subsequently.^13,14,15^ In the pre-ART era, patient survival was frequently poor.

In 2010, Stock et al. reported the outcomes of 150 prospectively enrolled, HIV-positive recipients of a renal transplant from HIV-negative donors.^16^ Recipient inclusion criteria included a cluster of differentiation 4 T-cell (CD4^+^) count ≥ 200 cells/µL and a suppressed viral load (VL) on a stable ART regimen prior to transplantation. Kidneys from both living and deceased donors were used. Patient survival rates at 1 and 3 years were approximately 95% and 88%, respectively, and graft survival was 90% and 74%, respectively. These percentages were lower than the national US average at the time, although they were comparable with results for other high-risk renal transplantation groups. Importantly, no evidence was seen of any immunosuppression-precipitated HIV viraemia, nor of any HIV-related opportunistic infections. Two patients developed limited cutaneous Kaposi’s sarcoma that was successfully treated, but no other sign of increased malignancies was observed in comparison with HIV-negative kidney transplant recipients.

Muller et al. then demonstrated the feasibility of renal transplantation from deceased HIV-positive donors to HIV-positive recipients. Initial results were reported in 2010, and long-term follow-up results in 2015.^17,18^ Among 27 patients, survival at 1, 3 and 5 years was 84%, 84% and 74%, respectively, and graft survival was 93%, 84% and 84%, respectively. Recipient inclusion criteria included a CD4^+^ count ≥ 200 cells/µL, a suppressed VL and ART duration > 3 months prior to transplantation. Patients with acquired immune deficiency syndrome (AIDS)-defining opportunistic infections and malignancies were excluded. The VL remained suppressed in all patients during the study period.

Following these data, an advocacy campaign in the USA led to the passage of the *HIV Organ Policy Equity (HOPE) Act* in 2013, which allowed for research into transplanting organs from HIV-positive donors into HIV-positive recipients.^19^ When this became federal policy in 2015, several US transplant centres began embarking on such efforts. To date, these have included deceased donor HIV-positive-to-HIV-positive kidney and liver transplantations, and living donor HIV-positive-to-HIV-positive kidney transplantations.^20,21,22^ Successful outcomes in heart, pancreas and lung transplants have also been reported in HIV-positive patients, despite using organs from HIV-negative donors.^23,24,25,26^

### Human immunodeficiency virus-negative recipients of solid organs from human immunodeficiency virus-positive donors

Prior to 2017, HIV-positive-to-HIV-negative transplantations both internationally and locally had been inadvertent (because of diagnosis of the donor’s HIV status subsequent to transplantation). In 2017, Botha et al. performed the first intentional liver transplantation from a living HIV-positive donor to an HIV-negative recipient.^27^ The recipient was a 7-month-old child with biliary atresia and end-stage liver disease who was placed on the waiting list for a liver transplant from an HIV-negative donor. After a prolonged period on the waiting list, the child’s HIV-positive mother requested to be considered as a donor because she was otherwise a suitable candidate and furthermore fulfilled donor criteria outlined in the *HOPE Act*. She was on stable ART, had a CD4^+^ count > 200 cells/µL and was virally suppressed with no evidence of any opportunistic infections or AIDS-associated malignancies. Following extensive multidisciplinary meetings and counselling, permission for the procedure was obtained from the local institutional review board as part of a research trial, and both of the child’s parents consented to the procedure. The recipient received triple ART before the transplantation to minimise the risk of HIV transmission, and this was continued after transplantation. To date, the recipient remains well, with normal-for-age growth and excellent graft function. HIV antibodies were detected at day 43 post-transplantation, although this response gradually attenuated with time. No plasma or cell-associated HIV-1 DNA or RNA was detected at any stage in the recipient, although early post-transplantation samples were not available for testing.

## Transmission risks

Blood from donor organs are routinely flushed out prior to insertion in the recipient. However, these organs may still contain replication-competent HIV virions. Small amount of donor blood may remain in the organ despite flushing. Furthermore, HIV can infect the renal tubular epithelial cells, podocytes and parietal epithelial cells of the kidney.^28,29,30^ Similarly, it is known that the liver’s Kupffer cells and sinusoidal endothelial cells may be infected by HIV, as are hepatoma cells.^31,32^ In addition, HIV could be transmitted by free virus or lymphocytes carried in the interstitium of the organ. Recent work suggests that HIV reservoirs probably persist in all deep tissues, although replication-competent viruses likely comprise only a minority of viral strains.^27,33,34^

### Human immunodeficiency virus-positive recipients

There is concern that, despite being on ART for at least several months at the time of transplantation, the HIV-positive recipient may be at risk of acquiring a second strain of HIV via residual virus in the allograft. This strain could either replicate independently or generate a new recombinant viral strain. Both are rare phenomena that have previously been documented in non-transplantation settings.^35,36,37,38^ Of critical concern would be the transmission of an HIV strain that is unlikely to be controlled with ART in the recipient, either because of extensive resistance or because of recipient contraindications to particular antiretroviral drugs. In theory, despite the flushing of blood, the risk of allograft transmission would be higher with organs from donors with unsuppressed VLs at the time of donation (such as might be seen in some deceased HIV-positive donors).

Selhorst et al. recently reported on the impact of the donor strain on the recipient’s HIV control in Muller’s HIV-positive-to-HIV-positive renal transplant cohort.^39^ Plasma and peripheral blood mononuclear cell (PBMC) samples were analysed from donor–recipient pairs. Donor virus was detectable in 8/25 recipients (32%) on deep sequencing in plasma samples taken between 1 and 6 weeks post-transplantation. Deep sequencing of PBMC samples, targeting reverse transcriptase and the *env* gene’s V3 region, found drug resistance mutations in a minority of both donors and recipients, but without clear evidence of any transmitted resistance from donor to recipient in 24/25 recipients. Possible superinfection was detected in one recipient from a sample taken 12 weeks post-transplantation. However, donor sequences were not found in the recipient’s PBMC samples taken before (6 weeks) or after (26 weeks) that, nor when the 12-week sample was sequenced again. It is unclear whether donor proviral sequences detected in this recipient in a single sample represent true superinfection or shedding of previously infected donor kidney cells into the blood. None of the recipients have to date failed ART post-transplantation.

Although somewhat reassuring, there are several limitations to these data. Only one of the donors had drug resistance mutations present at a level > 0.5% (levels thought to be physiologically relevant), and no plasma or PBMC samples from the recipient in that case were available for analysis. Furthermore, the recipient’s ART regimen in Muller’s cohort had substantial activity against the donor strain in each case; this may not be true of a patient cohort with more complicated pre-transplantation ART histories. In addition, because none of the donor patients were on protease inhibitors (PIs) or integrase strand transfer inhibitors (InSTIs) – only reverse transcriptase was sequenced for drug resistance mutations – the effect of mutations involving protease or integrase remains to be determined. Lastly, the ability to detect superinfection was limited by the low proviral loads found in the recipients (who were for the most part virally suppressed post-transplantation) and by anti-thymocyte globulin-induced T-cell depletion.

Most recently, Blasi et al. reported finding a donor’s HIV strain in an HIV-positive recipient’s blood and urine up to 16 days after kidney transplantation, but not thereafter.^33^ Importantly, the donor HIV strain was susceptible to the recipient’s ART regimen from the outset, though the recipient’s regimen was additionally fortified with rilpivirine (RPV) from postoperative day 1 as a precaution.

In summary, donor HIV viral strains appear to be detected in the blood of recipients in many cases within the first few weeks after transplantation, although whether this represents productive infection of new cells, lysing of donor-derived infected cells or a combination of both is unclear. Considering the potential risk of superinfection, we recommend that an infectious diseases and/or HIV expert should review all available donor HIV treatment history and resistance test information prior to transplantation, and that an anticipated inability of the recipient to control the donor HIV strain should be considered a contraindication to transplantation (see ‘Recommendations’ section).

### Human immunodeficiency virus-negative recipients

The risk of an HIV-negative recipient acquiring HIV from an organ from an HIV-positive donor is currently unknown, and is likely to be influenced by multiple donor and recipient characteristics, and the nature of the solid organ that is transplanted. When the donor’s HIV infection is only diagnosed subsequent to transplantation (because of the donor inadvertently being in the window period of HIV testing), HIV infection of the recipient appears highly likely. However, under the controlled conditions described by Botha et al., where the donor has a stably suppressed VL and the recipient is started on ART prior to transplantation, the risk of HIV transmission to the recipient is far less certain.^27^ In the one such published case to date, the results of HIV testing were equivocal, with initial seroconversion at day 43 and then slow waning of the serological response approaching undetectable levels over the course of a year. No plasma or cell-associated HIV has been detected in the recipient even by using ultrasensitive assays, although this testing was only possible on samples obtained from day 111 onwards. The interpretation of these results is not straightforward, particularly in the post-transplantation setting, where antibody responses may be attenuated because of anti-rejection immunosuppression. Possible explanations for the HIV antibody pattern observed include the recipients’ systemic infection with HIV, confinement of the HIV infection within the maternally derived donor liver, a purely serological response generated by the donor liver, and a recipient serological response generated to HIV antigens in the absence of replication-competent virus.^27,34,40^

We recommend that all available donor HIV treatment history and resistance test information should be reviewed prior to transplantation, as for HIV-positive recipients. Any anticipated inability of the recipient to control the donor’s HIV strain should similarly be a contraindication to transplant, regardless of the precautions put in place to limit the acquisition of HIV (see ‘Recommendations’ section).

## Recommendations for donor and recipient eligibility

### Recipient eligibility

#### Human immunodeficiency virus-positive recipient

Eligibility criteria for HIV-positive transplant recipients:

- CD4^+^ count ≥ 200 cells/µL (≥ 100 cells/µL can be considered for liver transplant recipients provided there is no history of opportunistic infections or malignancies). For children aged < 5 years, a CD4^+^% threshold of 15% should be used.
- Chronic patients: plasma VL < 50 copies/µL (most recent test performed within 3 months prior to transplantation).
- For organs from HIV-positive donors: the recipient must be able to tolerate an ART regimen effective against the donor’s HIV strain.

**Rationale:** Patients are required to have a CD4^+^ count ≥ 200 cells/µL (for patients aged < 5 years, a CD4^+^% threshold of 15% should be used). Although any cut-off is somewhat arbitrary, we endorse a CD4^+^ threshold of 200 cells/µL for two reasons: (1) it is a threshold which provides protection against many opportunistic infections, some of which may be difficult to diagnose and may cause significant post-transplantation morbidity and mortality; and (2) with the exception of liver transplants, the safety of organ transplantation below this recipient CD4^+^ level has not been established, as trials have generally excluded patients with CD4^+^ counts below this level. In the case of HIV-positive recipients of liver transplants, there is evidence that using a CD4^+^ threshold ≥ 100 cells/µL is safe provided there is no history of any opportunistic infection or malignancy (in which case a CD4^+^ threshold of 200 cells/µL is recommended).^41^ Another exception to the rule would be immune non-responders, who fail to reconstitute an adequate CD4^+^ count despite prolonged viral suppression, but this requires consultation with an infectious diseases specialist on a case-by-case basis.

The patient must also demonstrate virological control of their HIV, as evidenced by a suppressed plasma VL within the 3 months prior to transplantation. Achieving this demonstrates that the patient is able to tolerate and adhere to their ART regimen and provides sufficient time to unmask any immune reconstitution inflammatory syndrome (IRIS) reactions. In the setting of acute organ failure, however, patients may not yet have had sufficient time to obtain a suppressed VL, which may take 3 or more months. Patients on ART for shorter than this time period may still be considered for transplantation provided that their CD4^+^ count exceeds the thresholds above, but consultation with an infectious diseases specialist is advised. Such patients would be regarded as being at higher risk post-transplantation than patients demonstrating stable VL suppression.

Information about the donor’s ART history may not be available in certain time-sensitive transplantation scenarios, such as with deceased donors. Although every effort should be undertaken to obtain such information, transplantation should not be delayed unduly in its absence.

#### Human immunodeficiency virus-negative recipient

- The benefit of accepting an organ from an HIV-positive donor must outweigh the potential risks thereof and the risks of remaining on the transplant list while awaiting an organ from an HIV-negative donor.
- The recipient (and/or caregiver in the case of a minor) must receive appropriate counselling about the potential additional risks of the procedure given the donor’s HIV-positive status.
- The recipient must be able to tolerate an ART regimen effective against the donor’s HIV strain and must agree to take lifelong ART.
- Transplantation should be undertaken as part of a human research ethics committee (HREC)-approved research protocol.

**Suggested antiretroviral therapy:** The following ART is suggested for HIV-negative recipients of organs from HIV-positive donors:

- The regimen chosen will vary according to the donor’s ART history and the recipient’s comorbidities. For most recipients weighing > 20 kg, we suggest using a dolutegravir (DTG)-based regimen where possible. Dolutegravir has a very high barrier to resistance, is only rarely hepatotoxic and has no significant drug–drug interactions with commonly used immunosuppressant drugs. Dolutegravir is now freely available in both the public and private sectors. Regimens for paediatric patients weighing < 20 kg should be discussed with a paediatric HIV expert.
- We suggest starting ART prior to transplantation, so as to achieve therapeutic drug levels at the time of surgery. The exact time period required is not currently well defined, but commencing therapy 2–3 days prior to transplantation should provide adequate time for the drugs to achieve steady state at the time of transplantation (i.e. approximately 4 half-lives of the drug).
- We currently suggest continuing ART indefinitely. Any decision to stop ART at a later stage should only be made if, despite intensive testing (including with highly sensitive assays), there is still no clear evidence that HIV transmission took place, and this should only be undertaken after informed consent and with the approval of the HREC overseeing the case.

### Donor eligibility

#### Living human immunodeficiency virus-positive donor

- Standard living donor work-up
- Duration of ART ≥ 3 months
- CD4^+^ count ≥ 200 cells/µL (cluster of differentiation 4 T-cell percentage [CD4^+^%] ≥ 15% for patients aged < 5 years)
- Plasma VL < 50 copies/µL
- The recipient must be able to receive a safe and effective ART regimen, considering the donor’s anticipated HIV viral resistance strains.

**Rationale:** The prospective donor must have demonstrated durable and stable control of their HIV, to minimise the risk of unmasking IRIS reactions occurring subsequent to organ donation, which could jeopardise the health of the donor. A CD4^+^ threshold ≥ 200 cells/µL is also recommended to minimise the likelihood of occult opportunistic infections either manifesting after organ recovery or being transmitted to the donor during transplantation. We do not consider a -pre-transplantation biopsy of the donor organ to be a routine requirement merely because of HIV infection.

#### Deceased human immunodeficiency virus-positive donor

- Standard criteria, as for HIV-negative deceased donors.
- For deceased donors with a history of HIV resistance or virological failure, the recipient must be able to receive a safe, tolerable and effective ART regimen considering the donor’s known or inferred patterns of viral resistance.

**Rationale:** Although the risk of transmission of the donor virus to the recipient is likely to be higher if the donor has an unsuppressed VL, limiting the deceased donor pool only to virally suppressed individuals would significantly restrict the number of organs available. Deceased donors in South Africa had VLs that ranged from undetectable *to* > 150 000 copies/µL, and none of the recipients to date have developed virological failure from a transmitted strain of HIV.^39,42^ For donors with an unsuppressed VL, we recommend that the recipient should be placed on an ART regimen that would be expected to treat both their own and the donor’s HIV strains. Consultation with an infectious diseases specialist experienced in managing transplant patients is mandatory.

## Care of the human immunodeficiency virus-positive recipient after transplantation

For the most part, we recommend routine HIV care post-transplantation, with minor alterations where indicated:

- In view of the potential for unanticipated treatment interruptions and drug–drug interactions, we recommend performing an HIV VL measurement within 2–3 months after transplantation. A VL > 50 copies/µL should prompt urgent intervention as per the latest Southern African HIV Clinicians Society Guidelines.
- Key drug–drug interactions are outlined in Table 1. Physicians should be aware of these and consider changing therapy accordingly if required. In general, InSTI-based ART offers the fewest drug–drug interactions with commonly used immunosuppressant drugs, as well as a high barrier to resistance.
- HIV-positive transplant recipients should receive the same vaccines as HIV-negative transplant recipients.
- HIV-positive transplant recipients should receive the same post-transplantation prophylaxis for opportunistic infections as HIV-negative transplant recipients.

**TABLE 1:** Key drug–drug interactions between commonly used immunosuppressant and antiretroviral drugs.

Variable	EFV	*RPV*	*PIs*	*DTG*
*Calcineurin inhibitors*	Small decrease in tacrolimus level	No change	Calcineurin inhibitor level severely raised	No change
*mTOR inhibitors*	Moderate decrease in mTOR inhibitor level	No change	mTOR inhibitor level severely raised	No change
*Prednisone*	No change	No change	Prednisone level moderately increased	No change

DTG, dolutegravir; EFV, efavirenz; mTOR, mammalian target of rapamycin; PIs, protease inhibitors; RPV, rilpivirine.

### Ethical consideration

In all cases, the decision to receive an organ from an HIV-positive donor should be made freely and without coercion. In addition, potential organ recipients should be made aware of the possibility of receiving an organ from an HIV-negative donor. In the case of children who received HIV-positive donor organs and have not reached the age of consent, every effort must be made to ensure the protection of their best interests.

Adult recipients of organs from HIV-positive donors and caregivers of child recipients must be made aware of the importance of adherence to antiretroviral medication, and the complexities inherent in this type of transplant. To this end, a social worker should be an integral part of the transplant team involved in donor and recipient assessment, and this person should be in a position to empower potential recipients, and their caregivers, in a manner that will facilitate favourable outcomes.

### Living human immunodeficiency virus-positive donor

In addition to standard donor criteria, it is important that the HIV-related clinical criteria outlined below (see also ‘Recommendations’ section) are adhered to, so as to minimise harm to the donor. This may be especially important if the donor is a close relative or friend of the recipient, where conflicts of interest may arise.

We advise that an independent donor advocate (IDA) should be appointed for all cases involving a living HIV-positive donor. An IDA is a person with a good understanding of transplants, who is fully independent of the donor, the recipient and the medical team; they need not be a health professional. The role of an independent advocate is to ensure that the donor’s interests and rights are upheld at all times, and to ensure that the donor has adequate understanding of the consent process, surgical procedure and follow-up requirements. Independent donor advocates should be a required signature on the surgical consent form, affording them veto status for the procedure. Although IDAs are a legal mandate of most living donor transplant programmes in many countries, this is not currently the case in South Africa. However, we regard an IDA as essential for any programme using increased-risk living donors, including living HIV-positive donors.

### Human immunodeficiency virus-positive recipients

It is a key principle of medical ethics that equal access to treatment should not be denied unreasonably. Where outcomes in HIV-positive recipients of organs have been shown to be similar to other patient groups who are offered organ transplantation, as with renal transplants, HIV status alone cannot be used as grounds for exclusion from transplant programmes. Where outcomes for HIV-positive organ recipients are not known, it should not be assumed that HIV-positive recipients will necessarily fare more poorly than other transplantable groups. Rather, well-monitored clinical trials are encouraged to ascertain outcome data. Increasingly, survival data from HIV-positive recipients of solid organs other than kidneys are also proving similar to those of HIV-negative controls in many instances, although often with an increased risk of rejection.^23,25,34^

As with any disease, medical complications of a condition may legitimately disqualify patients from transplantation. In the case of HIV, these may include active opportunistic infections or AIDS-associated malignancies. However, patients with HIV should not be disadvantaged solely on the basis of their HIV.

### Human immunodeficiency virus-negative recipients of organs from human immunodeficiency virus-positive donors

Currently, it is not definitively known whether, and at what frequency, HIV is transmitted from the donor organ to an HIV-negative recipient in a controlled environment when attempts are made to limit transmission. In the absence of definitive data, it is prudent to assume for ethical purposes that this likelihood may be substantial. Extreme care should therefore be taken to ascertain that the risk of acquiring HIV is outweighed by the risk of continuing to wait for a transplant from an HIV-negative donor. We anticipate that further data on HIV’s transmissibility in these scenarios may inform these ethical considerations.

All potential transplant recipients in this situation should be informed fully of the potential that they might acquire HIV infection, and that the treatment for this will likely require lifelong ART.

#### Considerations for minors

Minors who are HIV-negative recipients of organs from HIV-positive donors require special consideration. This scenario may be particularly frequent for living-donor liver recipients, who are most commonly children because of organ size considerations. Given that we recommend that transplantations involving HIV-positive patients be performed under the review of a local research ethics committee (see below), the *South African National Health Act* requires consent from the minor’s primary caregiver for the procedure regardless of the minor’s age. When the minor is capable of understanding the procedure, the minor’s assent should also be sought.

Additional ethical considerations for minors include:

- *The capacity of the child’s support network to cater for the additional burden of HIV-related therapies and potential complications*: The child will require extensive assistance in the post-transplantation period, and this may include ART, additional clinic visits to optimise HIV control and additional admissions in the case of opportunistic infections.
- *The need for age-appropriate disclosure to the child of their HIV status should transmission occur*: Best practice principles in this regard have been established within the HIV field, and include serial disclosures by qualified counsellors in the presence and with the support of the child’s primary caregivers (usually the parents), at a complexity level appropriate for the child’s understanding at that age.
- *Donor disclosure*: HIV status disclosure facilitates adherence, and adherence in transplant programmes is essential to promoting good outcomes. It is strongly encouraged that the primary caregiver of the potential recipient child (often the mother, who may also be the donor) has disclosed her or his HIV status to her or his immediate support ‘network’ who will be involved in caring for the recipient child in future. This network may be immediate family members, or it may be a family member at a distant location.

### Research protocols and processes

Given the rapidly developing nature of the field, and the ethical and medical complexities involved, we advise that organ transplants involving HIV-positive patients should be subject to formal ethics review by a local HREC. Rigorous patient protections are particularly vital in the case of HIV-negative recipients, given the ethical considerations involved. We recommend involvement of the local HREC as early as possible in the process, to allow adequate time for discussions and detailed interactions.

Furthermore, we strongly recommend that all data concerning transplantations with HIV-positive donors or recipients should be peer-reviewed, published and distributed timeously because the experiences shared from other centres may have biomedical and ethical implications.

## Conclusion

These guidelines aim to sketch an evidence-based framework for HIV-positive donors and recipients regarding solid organ transplantation. It is expected that specific recommendations will need to be adjusted as further evidence becomes available. Furthermore, individual patient circumstances may require deviations from this document, provided that such departures have the backing of the local research ethics committee and contribute to continuing research in the field. These guidelines aim to provide support and a framework for the expansion of programmes incorporating HIV-positive donors and recipients.
